# 肺移植治疗肺癌1例报告并文献复习

**DOI:** 10.3779/j.issn.1009-3419.2011.07.14

**Published:** 2011-07-20

**Authors:** 烨铭 王, 静瑜 陈

**Affiliations:** 214023 南京，南京医科大学附属无锡市人民医院胸外科、肺移植科 Department of General Thoracic Surgery, Department of Toracic Transplant, Wuxi People's Hospital Affiliated to Nanjing Medical University, Wuxi 214023, China

**Keywords:** 肺移植, 肺肿瘤, 细支气管肺泡癌, 预后, Lung transplantation, Lung neoplasms, Bronchioloalveolar carcinoma, Prognosis

## Abstract

**背景与目的:**

肺癌被认为是肺移植的相对禁忌症，但随着移植技术的进步，此类非常规疾病也被纳入肺移植适应症。本文通过报道1例双肺移植成功治疗双侧肺癌男性患者的病例，并结合相关文献复习，探讨肺移植治疗肺癌的适应症及疗效。

**方法:**

2010年10月21日南京医科大学附属无锡市人民医院肺移植组为1例42岁男性患者成功进行了序贯式双肺移植，该患者术前胸部CT及PET-CT示双肺多发斑片状及团块样浸润阴影，纵隔淋巴结不肿大，其余部位未见转移。术后病理证实为粘液型细支气管肺泡癌，分期T4N0M0，Ⅲb期。

**结果:**

患者术后予常规三联免疫抑制，抗细菌、真菌、病毒等一系列治疗，并于术后66天恢复良好出院并定期随访，术后6个月随访时肺功能良好，未见明显转移征象。

**结论:**

对于某些合适的肺癌患者，肺移植是一个有效的治疗方法。

## Abstract (en)

**Background and objective:**

Lung carcinoma is a relative contraindication for lung transplantation (LTx). The improvement of this procedure may successfully treat novel end-stage pulmonary diseases. In the present study, we reported one case of bilateral LTx (BLT) in a young man with bilateral lung carcinoma and reviewed related literature to investigate patient selection and curative effect.

**Methods:**

A 42-year-old male patient underwent BLT on October 21, 2010 in our hospital. Preoperative chest CT and PET-CT showed bilateral multiple nodules and mass without positive lymph nodes or extrapulmonary metastasis. Bronchioloalveolar carcinoma was identifed histologically (T4N0M0, stage Ⅲb).

**Results:**

Routine therapies were performed postoperatively. The patient was discharged on post-operative day 66. Tere was no clinical or radiologic evidence of the recurrence of lung carcinoma in his latest follow-up of 6 months postoperatively.

**Conclusion:**

LTx may be proposed as an efcient therapeutic option in selected patients of lung carcinoma.

肺癌易远处转移，且肺移植术后由于免疫抑制剂的应用，患者术后仍有较大风险复发和转移，因而肺癌一向不作为肺移植的适应症^[1]^。2010年10月21日南京医科大学附属无锡市人民医院肺移植科为1例双侧细支气管肺泡癌的男性患者进行了序贯式双肺移植，手术效果良好。现将临床资料总结报道如下，并行文献复习，探讨肺移植治疗肺癌的适应症及疗效。

## 1 临床资料

受体资料：男性患者，42岁，身高172 cm，体重45kg，“A”型血，曾因右肺癌于2008年8月行右中下肺叶切除术，术后病理提示粘液型细支气管肺泡癌，支气管旁（0/5）、第3组（0/6）、第7组（0/5）、第9组（0/1）及第11组（0/2）淋巴结均未见癌转移，分期Ⅲb期（T4N0M0），肿瘤组织EGFR基因检测未见突变，*K-ras*突变。术后患者予GP方案化疗4次。1年后，再次出现咳嗽、咳痰症状，在2009年9月复查PET-CT示右上肺叶及左全肺多发斑片状及团块样浸润阴影，FDG代谢增高，纵膈淋巴结无肿大，其余部位未见转移（图 1A）。患者于2009年9月-12月期间接受厄洛替尼靶向治疗，后又予培美曲塞全身静脉化疗2次，但治疗后患者症状无明显变化，多次复查胸部CT示肺内病灶有明显增大趋势（图 1B）。在其它治疗均无效的情况下，患者于2010年9月27日来我院进行肺移植评估，术前胸片及CT示：右胸腔缩小，右横膈上抬，右肺野及左上下肺野大片团块浸润阴影，左胸腔大小正常。再次查PET-CT仍未发现肺外转移征象。术前心脏彩超提示左室射血分数为62%，无三尖瓣返流，肺动脉压正常；肺功能提示用力肺活量（forced vital capacity, FVC）及一秒钟用力呼气容积（forced expiratory volume in one secord, FEV_1_）分别为1.93 L及1.1 L（分别占预计值的36.5%及32%）；同时，血气分析显示Ⅱ型呼吸衰竭（PaO_2_ 52 mmHg, PaCO_2_ 56 mmHg）。

**1:**
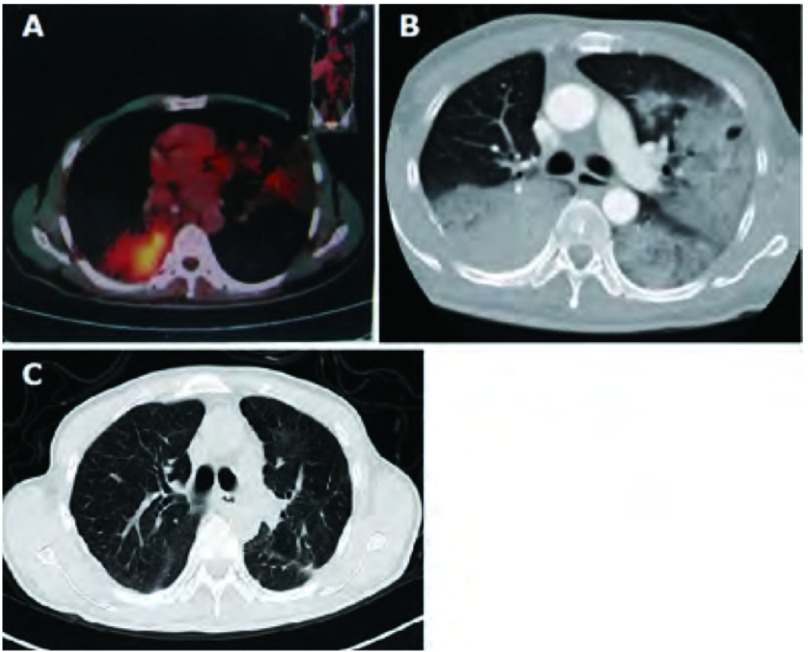
患者临床影像学特征。A：术前PET-CT示双肺多发斑片状及团块样浸润阴影，纵隔淋巴结不肿大，其余部位未见转移；B：术前胸部CT示双肺野多发大片状密度增高影，边缘模糊，左肺见多个结节影；C：术后6个月随访复查胸部CT显示双肺复张良好，未见明显肿瘤复发。 Clinical radiologic features of the patient. A: Preoperative PET-CT showing bilateral multiple nodules and mass without positive lymph nodes or extrapulmonary metastasis; B: Preoperative chest CT scan showing diffuse bilateral ground-glass opacities and nodules; C: CT scan of the chest obtained 6 months postoperative showing satisfactory reexpansion of double lungs with no evidence for recurrence.

供者资料：男性，28岁，身高175 cm，体重75 kg。供、受体血型相符，供肺的维持、获取及保存见文献^[2]^。

手术及术后情况：患者于2010年10月21日在麻醉、气管插管后，经双侧前胸切口不横断胸骨完成非体外循环下序贯式双肺移植^[3]^。手术先行右侧前外侧切口开胸，分离胸壁、肺门粘连，心包内游离右肺动脉、右上肺静脉及右下肺静脉残端，后切除右上肺叶，植入右全肺，因右侧胸腔较小，右全肺膨胀后无法完全纳入右胸腔，肺扩张后部分肺暴露在胸腔外，维持患者氧合。同法打开左胸，左肺无粘连，顺利切除左病肺。植入左全肺后，首先关左胸，然后依据右胸腔大小将植入的右全肺行减容，切除右下肺叶后关胸。手术顺利完成，全程6.5 h，右肺、左肺冷缺血时间分别为4 h和6 h。术后病理仍为粘液型细支气管肺泡癌，左肺支气管旁（0/9）、左第5组（0/2）、左第10组淋巴结、右第4组（0/3）、第7组淋巴结（0/3）均未见癌转移。术后采用他克莫司+吗替麦考酚酯+类固醇激素的免疫抑制方案，ICU监护、呼吸机脱机原则及控制感染等见报告^[4]^。术后第4天左肺再灌注损伤，予ECMO辅助治疗3 d后撤离。术后两周又发生一次左侧自发性气胸，予积极手术治疗后好转，于术后66 d康复出院。术后6个月检查肺功能良好，胸部CT未发现肿瘤复发迹象（图 1C），目前随访中。

## 2 讨论

世界上首例人体肺移植在1963年由Hardy等^[5]^完成，受体是1例IIIb期的肺癌患者，然而，由于当时移植技术的缺陷，这名患者术后18 d死于肾衰竭。直到1983年，多伦多肺移植团队成功完成首例长期生存的肺移植^[6]^，20多年来，肺移植日益成为治疗终末期肺病的有效手段。在其它移植领域，例如肝细胞癌已成为肝移植治疗的常规手术适应症^[7]^。肺癌目前被认为是肺移植的相对禁忌症，而细支气管肺泡癌（bronchioloalveolar carcinoma, BAC）的肺移植，目前病例逐渐增多^[8]^。

有统计数据^[9]^显示，5%的非小细胞肺癌（non-small cell lung cancer, NSCLC）为BAC，而混合型（BAC+腺癌）则占约20%，两种类型的肺癌临床表现较为相似。30%的BAC患者常无吸烟史，远比鳞癌（5%）及腺癌（15%）高。双肺弥漫性BAC预后极差，中位生存期仅为4个月。目前对于BAC主要是采用手术、化疗及分子靶向治疗等的综合治疗^[9, 10]^，对于孤立病灶，一般采取楔形切除或肺叶切除；对于多发病灶，也可尝试多个楔形切除及肺叶切除，辅以表皮生长因子受体酪氨酸激酶抑制剂（epidermal growth factor receptor tyrosine kinase inhibitor, EGFRTKI）及化疗药物的应用是否改善其预后，仍不甚明了；而对于弥漫性BAC，一般采用铂类为基础的化疗、EGFR-TKI靶向治疗及肺移植^[9]^。Paloyan等^[11]^发现，BAC虽然肺内转移极为迅速，但是很少出现肺外转移，因此他们认为这可以做为肺移植治疗BAC的依据，对于一些不能手术切除治愈或是复发性的BAC，肺移植不失为一个有效的治疗方法。然而，国外供体相对短缺，肺移植治疗肺癌的病例较少^[12]^。

1991年，Etienne等^[13]^为一例罹患BAC的女性患者行双肺移植术，术后长期随访5.5年无肿瘤复发，这是第一例肺移植成功治疗BAC且长期存活的报道；Garver等^[14]^在1999年报道了7例肺移植治疗BAC的病例，4例（3例双肺、1例单肺）在术后10个月-48个月复发，另外3例（2例双肺、1例单肺）在随访期间未出现复发。对其中3例出现肿瘤复发的患者，通过PCR分别检测其供肺、原发肿瘤及复发肿瘤发现，复发肿瘤起源于受体。因此他们认为，虽然BAC在术后易出现复发，但肺移植治疗BAC在技术上是可行的，单、双肺移植均可作为治疗手段。Zorn等^[15]^报道9例肺移植（2例单肺，7例双肺）治疗BAC，5年生存率为52%，死亡原因为移植肺肿瘤复发导致的呼吸衰竭。

2004年的一篇文献^[1]^对全球主要肺移植中心的数据进行汇总，并回顾性分析26例BAC的肺移植，发现：①BAC患者术后远期存活率接近国际心肺移植协会统计资料的平均水平。术后5年及10年存活率，ISHLT在2003年的统计数据分别为45%、23%，相比之下，BAC患者则为39%及31%。26例接受肺移植治疗的BAC患者中，4例早期死亡，分别为原发性移植物失功2例，右心衰1例，心源性休克1例。存活的22例中，有13例于术后5个月-49个月（中位12个月）肿瘤复发，其中9例死于术后11个月-82个月（中位22个月）。②Ⅰ期肺癌患者术后存活率接近甚至高于ISHLT平均水平，Ⅱ期和Ⅲ期肺癌患者预后相对较差。在随访过程中（3个月-120个月，中位30个月），22名Ⅰ期患者有14例未出现复发，5年存活率达51%；而在14例Ⅱ期和Ⅲ期患者中，有9例于术后4个月-16个月（中位8个月）死于肿瘤复发，仅有2例分别在术后20个月和98个月随访时仍健在。

此外，除了无肺外转移的原发性肺癌，国外亦有肺移植治疗肺部转移性肿瘤的成功经验。Shargall等^[16]^报道多伦多肺移植团队为1例43岁、有“子宫纤维瘤”手术史、复发“良性转移性平滑肌肉瘤”的女性患者行双肺移植术，该患者仅发现肺内转移，而无他处肺外转移。这名患者在移植术后20个月随访时仍未发现复发迹象。这是全球首例应用肺移植治疗肺部转移性肿瘤的病例。患者预后良好，其经验值得我们借鉴。

本文报道的病例的病理诊断为粘液型细支气管肺泡癌，曾有右中下肺叶切除手术史，并多次化疗及靶向治疗，后因肿瘤复发转移导致呼吸衰竭，在患者强烈要求下而纳入肺移植名单。术前各项检查并未发现有肺外转移征象，因此这也成为我们为其行双肺移植手术的依据。患者术后66 d出院，在术后6个月随访时肺功能良好，尚未发现肿瘤复发迹象，但患者远期生存仍需进一步随访。

总之，我们认为，肺癌可以作为肺移植的相对适应症，对于终末期肺病伴肺癌、原发性肺癌（包括BAC）及某些肺部转移性肿瘤均可考虑肺移植评估。这类患者术后因为免疫抑制剂的应用，仍有较大复发转移的风险，对肺癌患者的肺移植仍有待进一步研究。
